# 3,3′-Diindolylmethane Suppresses the Growth of Hepatocellular Carcinoma by Regulating Its Invasion, Migration, and ER Stress-Mediated Mitochondrial Apoptosis

**DOI:** 10.3390/cells10051178

**Published:** 2021-05-12

**Authors:** Suvesh Munakarmi, Juna Shrestha, Hyun-Beak Shin, Geum-Hwa Lee, Yeon-Jun Jeong

**Affiliations:** 1 Laboratory of Liver Regeneration, Biomedical Research Institute of Jeonbuk National University Hospital, Jeonju 54907, Korea; sanghzain@gmail.com; 2 Alka Hospital Private Limited, Jwalakhel, Kathmandu 446010, Nepal; zhun12an@gmail.com; 3 Department of Surgery, Jeonbuk National University Hospital, Jeonju 54907, Korea; no1kal@naver.com; 4 Department of Pharmacology and New Drug Development Research Institute, Jeonbuk National University Hospital, Jeonju 54907, Korea; vitamin2635@naver.com

**Keywords:** ER stress, unfolded protein response, hepatocellular carcinoma, apoptosis, EMT, DIM

## Abstract

Hepatocellular carcinoma (HCC) is the leading cause of cancer-related death worldwide with limited treatment options. Biomarker-based active phenolic flavonoids isolated from medicinal plants might shed some light on potential therapeutics for treating HCC. 3,3′-diindolylmethane (DIM) is a unique biologically active dimer of indole-3-carbinol (I3C), a phytochemical compound derived from Brassica species of cruciferous vegetables—such as broccoli, kale, cabbage, and cauliflower. It has anti-cancer effects on various cancers such as breast cancer, prostate cancer, endometrial cancer, and colon cancer. However, the molecular mechanism of DIM involved in reducing cancer risk and/or enhancing therapy remains unknown. The aim of the present study was to evaluate anti-cancer and therapeutic effects of DIM in human hepatoma cell lines Hep3B and HuhCell proliferation was measured with MTT and trypan blue colony formation assays. Migration, invasion, and apoptosis were measured with Transwell assays and flow cytometry analyses. Reactive oxygen species (ROS) intensity and the loss in mitochondrial membrane potential of Hep3B and Huh7 cells were determined using dihydroethidium (DHE) staining and tetramethylrhodamine ethyl ester dye. Results showed that DIM significantly suppressed HCC cell growth, proliferation, migration, and invasion in a concentration-dependent manner. Furthermore, DIM treatment activated caspase-dependent apoptotic pathway and suppressed epithelial–mesenchymal transition (EMT) via ER stress and unfolded protein response (UPR). Taken together, our results suggest that DIM is a potential anticancer drug for HCC therapy by targeting ER-stress/UPR.

## 1. Introduction

In the overall rate of cancer incidence and mortality, HCC is the fifth most common form of cancer and the third leading cause of cancer-related death worldwide. It is associated with an extremely poor prognosis [1,2]. Gradual increases of chronic liver disease and cirrhosis due to viral hepatitis, metabolic disorders, and alcohol intake are important risk factors for the development of HCC worldwide [3,4]. Due to its strong invasion ability and the complexity of the metastatic process, treatment options for HCC patients are very limited and its prognosis is dire [5,6]. Although there is a variety of therapeutic options such as chemotherapy, immunotherapy, intravenous drug embolization, and surgery in the treatment of HCC patients [7], many of them remain challenging and ineffective due to cytotoxicity and multidrug resistance. Therefore, developing an anticancer drug from plant-derived compounds as potential therapeutics for HCC is urgently needed.

The endoplasmic reticulum (ER) is a major site for regulating multiple cellular events such as proper protein folding and synthesis, post-translation modification, calcium regulation, and redox balance. Disturbance of these events can lead to accumulation of unfolded and misfolded proteins in the ER which is termed ER stress [8,9,10]. Recent studies have shown that ER stress plays crucial role in various diseases including cancer, metabolic disorders, autoimmune disorders, and neurodegenerative disease [11,12]. Endoplasmic reticulum (ER) stress plays a defensive role in maintaining cell survival in response to various pathophysiological factors triggered by the activation of PKR-like endoplasmic reticulum kinase (PERK), unfolded protein response (UPR), inositol-requiring enzyme 1α (IRE1α), and activating transcription factor-6 (ATF-6) [13,14]. Previous studies have suggested that the induction of ER stress and the activation of UPR can compromise the development of phenotypic transition and apoptosis of epithelial cells [15,16]. Besides, ER stress can induce an overproduction of reactive oxygen species (ROS), resulting in the initiation of the intrinsic apoptotic pathway due to an imbalance of redox homeostasis [17,18]. Mitochondria are connected with the ER membrane by a unique region termed mitochondria-associated ER membrane (MAM) which is important for lipid metabolism, mitochondrial fission, autophagosome formation, apoptosis progression, and Ca^2+^ transport between the ER and mitochondria [19,20,21]. As far as cancer biology is concerned, activation of ER stress can release an excessive amount of Ca^2+^ to the mitochondria, causing loss of mitochondrial membrane potential (ΔΨm) and overproduction of ROS which can induce ER stress-mediated mitochondrial apoptotic pathway and suppress tumor growth [21,22,23]. Therefore, inducing ER stress and UPR activation has been considered as a potential therapeutic strategy for cancer treatment.

In cancer progression, epithelial–mesenchymal transition (EMT) begins with the loss of epithelial cell polarity, transformation into mesenchymal cells, followed by upregulation of N-cadherin, Vimentin, Snail, Slug, Smad 2/3, Twist, and Zeb1/2 as well as matrix metalloproteinase (MMP) expression, whereas inhibits the expression of the epithelial marker, E-cadherin [24,25]. During HCC development, EMT plays a crucial role in cancer progression, growth, and metastasis by acquiring malignant features such as migratory and invasion, stemness, and drug resistance [26,27]. Numerous shreds of evidence have suggested that provoking ER stress and activating UPR are involved in the expression of transcription factors responsible for the progression of EMT in several types of cells. In gastric cancer cells, UPR can enhance EMT under a chronic hypoxic condition [28]. XBP1 can potentiate EMT and invasion of breast and liver cancer cells by inducing snail expression [29,30]. ER stress can induce EMT in alveolar epithelial cells through Src-dependent pathways [31]. However, the involvement of crosstalk between ER stress and EMT in HCC remains unclear. Therefore, the present study aims to understand the importance of ER stress and EMT in HCC.

**Figure 1:**
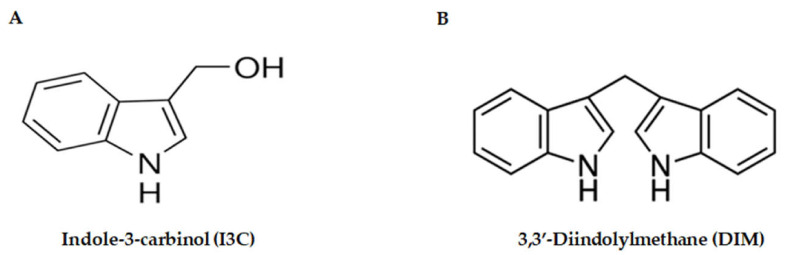
Molecular structures of flavonoids. (**A**) The molecular structure of indole-3-carbinol (I3C). (**B**) The molecular structure of 3,3′-diindolylmethane (DIM). DIM is a dimeric bioactive product produced by the major acid-catalyzation condensation of indole-3-carbinol (I3C) found in cruciferous vegetables.

Over the past few decades, several studies have explored therapeutic effects of plant-derived phytochemicals against cancers by regulating several molecular and metabolic processes [32,33]. 3′3-Diindolylmethane (DIM) is one potential phytochemical compound derived from acid-catalyzed condensation of indole-3-carbinol (I3C) found in Brassica species of cruciferous vegetables (broccoli, cabbage, and cauliflower) [34,35,36]. The molecular structure of DIM and I3C were shown in Figure 1A,B. Previously, numerous studies have shown that DIM can exert antitumor properties by inducing apoptosis and inhibiting proliferation, adhesion, migration, and invasion in different types of cancer, including HCC [35,37], breast cancer [38], esophageal cancer [39], colorectal cancer [40], prostate cancer [41,42], and pancreatic cancer [43]. However, the molecular mechanism involved in the effect of DIM on HCC remains controversial. We performed this study to determine the potential efficacy of DIM for decreasing cancer risk and/or for enhancing the effect of therapy on HCC.

In this study, we evaluated molecular mechanisms of anti-cancer effects of DIM in two HCC cell lines, Hep3B and HuhOur findings demonstrated that DIM could dose-dependently reduce proliferation, invasion, and migration of HCC cells and induce apoptosis by regulating ER stress and mitochondrial dysfunction of HCC cells. Our findings suggest that DIM can increase ER stress in HCC and result in the release of Ca^2+^, causing mitochondrial dysfunction and inducing apoptosis of HCC cells. Taken together, these results suggest that DIM is a potential anti-cancer therapeutic agent for treating HCC and other cancers.

## 2. Materials and Methods

### 2.1. Cell Culture and Reagents

Hepatocellular carcinoma cell lines Hep3B (HB-8064) were purchased from the American Type Culture Collection (ATCC, Manassas, VA, USA) and Huh7 (KCLB60104) cells were acquired from the Korean Cell Line Bank (KCLB, Seoul, South Korea). ATCC short tandem repeats (STR) were conducted systematically for cell line authentication. Cells were routinely monitored for mycoplasma contamination. Both cell lines were cultures in Dulbecco’s modified Eagle’s medium (DMEM; Hyclone^®^, Thermo Fisher Scientific, Inc., Logan, UT, USA) supplemented with 10% fetal bovine serum (FBS; Hyclone^®^, Thermo Fisher Scientific, Inc., UT, USA) and 1% penicillin-streptomycin (Hyclone^®^, Thermo Fisher Scientific, Inc., UT, USA) followed by incubation at 37 °C in a humidified incubator containing 5% CODIM was purchased from Sigma-Aldrich CO. (St. Louis, MO, USA). It was dissolved in DMSO at a final concentration of 10 mg/mL and stored at −20 °C until use.

### 2.2. Determination of Cell Proliferation

Cell proliferation was determined using MTT assay. Briefly, Hep3B and Huh7 cells were seeded into 96-well cell culture plates at a cell density of 5 × 10^3^ cells per well in 100 µL culture medium and allow to attach for 24 h. These cells were then treated with the indicated concentration of DIM (0, 20, 40, 60, 80, 120 µM) for 24 h. After 24 h of incubation, medium was removed and cells were treated with 50 µL of 50 mg/mL 2,5-diphenyl tetrazolium bromide (MTT; Sigma-Aldrich CO, St. Louis, MO, USA) for 3–5 h in dark. Then 100 µL of dimethyl sulfoxide (DMSO; Sigma-Aldrich CO, St. Louis, MO, USA) was added to dissolved transformed purple formazan crystals. The intensity of observed color was measured at a wavelength of 570 nm.

### 2.3. Clonogenic Formation Assay

To determine the inhibitory effect of DIM on colony formation of Hep3B and Huh7 cell lines, colony formation assays were performed. In brief, Hep3B and Huh7 cells were seeded in a 6-well plate at a density of 1000 cells per well and grown for 24 h. Cells were then treated with indicated concentration (20, 40, and 60 µM) of DIM in a fresh medium for 24 h. After 24 h, the medium was aspirated and replaced with a fresh DIM-free medium. Cells were allowed to grow for 10–14 days. Afterward, cells were fixed with ice-cold methanol for 15 min, washed with PBS, and stained with 1% crystal violet solution (Bioworld Tech. Inc., Bloomington, MN, USA), and incubated for 1 h at room temperature. Plates were washed with tap water and dried. Colonies having less than 10 cells were then counted using a densitometric software as described previously [44].

### 2.4. FACS Analysis

Apoptosis of HCC cells was evaluated using a propidium iodide/annexin V-FITC apoptosis detection kit (BD Biosciences, San Diego, CA, USA) according to the manufacturer’s instructions. Briefly, Hep3B and Huh7 cells were seeded into a 60-mm culture dish at a density of 5 × 10^4^ cells/well and allowed to attach for 24 h. These cells were then incubated with a fresh medium containing DIM at different concentrations (0, 20, 40, 60, and 80 µM) for an additional 24 h. Cells were harvested, washed with PBS and incubated with PI (5 µg/mL) for 5 min followed by the addition of Annexin V-FITC dye. After incubating in the dark at room temperature for 15 min, cells were analyzed using a flow cytometer.

### 2.5. Measurement of Fluorescent Ca^2+^ Release

Changes in [Ca^2+^] levels were measured as described previously [45]. Briefly, Hep3B and Huh7 cells were cultured at 70% confluency and then treated with DIM at indicated concentrations. Cells were harvested and incubated with 5 µM Fura-2 AM in culture medium for 1 h at 37 °C. Changes in [Ca^2+^] levels were evaluated by recording changes in Fura-2 ratio (F340/F380) with the addition of 5 µM thapsigargin, using the real-time mode of a PTI system (PTI Delta Ram, New Brunswick, NJ, USA).

### 2.6. Determination of Mitochondrial Membrane Potential (ΨΔm)

Mitochondrial membrane potentials (ΨΔm) of Hep3B and Huh7 cells were determined using tetramethylrhodamine ethyl ester perchlorate (TMRE) dye as described previously [4]. Briefly, Hep3B and Huh7 cells were treated with DIM (0, 40, 60, and 80 µM) for 24 h and stained with TMRE (400 nmol/L) for 20 min. Loss of mitochondrial membrane potential was determined by measuring the intensity of the TMRE dye at excitation and emission wavelengths of 549 nm and 575 nm, respectively, using a fluorescence plate reader.

### 2.7. Measurement of Reactive Oxygen Species (ROS) Levels

Intracellular ROS levels in Hep3B and Huh7 cells were determined using a fluorescent dihydroethidium (DHE) probe and an OxiSelect In Vitro ROS/RNS Assay Kit (Cell BioLabs, San Diego, CA, USA). To analyze ROS using the DHE probe, cells were stained with 5 µM of DHE, labeled with 4′,6-diamidono-2-phenylindole, dihydrochloride (DAPI), and examined under a fluorescence microscope using a ×63 oil immersion objective lens as described previously [4]. To determine the amount of ROS produced using the OxiSelect In vitro ROS/RNS Assay Kit following the manufacturer’s instruction, Hep3B and Huh7 cells were harvested after DIM treatment. Cell lysates were then prepared by resuspending 1 × 10^7^ cells in PBS with 0.5% NP40 (Sigma-Aldrich CO, St. Louis, MO, USA). The accumulation of ROS in HCC cells was calculated by monitoring the fluorescence intensity at excitation and emission wavelengths of 480 nm and 530 nm, respectively, with a SpectraMax Gemini XS Flouroimeter.

### 2.8. Transwell Cell Migration Assay

The ability of HCC cells to migrate was evaluated using a Boyden chamber assay. Briefly, Hep3B and Huh7 cells were seeded into the upper compartment of Boyden chambers (8-µm; Corning, NY, USA) containing polycarbonate membrane at a density of 5 × 10^4^ cells per well in 200 µL of serum-free medium containing DIM at indicated concentration. Then 500 µL of complete media were added to the lower compartment. After 24 h of incubation, the suspension was discarded and cells at the upper surface of the chamber were removed with cotton swabs. Migrated cells into the lower membrane surface were stained with a Diff-quick solution. Migrated cells were imaged under a light microscope. Three random areas were counted to calculate the average number of migrated cells per area.

### 2.9. Transwell Cell Invasion Assay

The invasion ability of HCC cells was evaluated using Corning^®^ Matrigel^®^ Invasion Chambers (8-µm; Corning, NY, USA) coated with a basement matrix. HCC cell lines Hep3B and Huh7 were seeded in Matrigel coated upper chambers at a density of 4 × 10^4^ cells per well in 200 µL serum-free medium containing DIM (0, 20, 40, and 60 µM) whereas 750 µL 20% FBS supplied medium was added to the lower chamber and incubated for 24 h. The suspension from the upper chamber was discarded and cells were removed by wiping them off the top membrane with cotton swabs. Cells that invaded lower membranes were then fixed with 100% ice-cold methanol for 30 min and stained with the Diff-Quick kit solution. The percent of invaded cells was counted from three randomly chosen areas under a light microscope.

### 2.10. Immunoblotting

Changes in protein expression level in response to DIM treatment were determined by Western blot analysis. Methods of protein extraction, quantification, and Western blotting were mentioned previously [4,46]. Proteins were transferred from gels onto PVDF membranes and incubated with primary antibodies overnight. Specific primary antibodies and horseradish peroxidase-conjugated secondary antibodies were used in Western blot. They are listed in Table 1. Blots were washed with TBST. Protein signals were enhanced and visualized using a chemiluminescence detection system.

### 2.11. Statistical Analysis

All experimental results are from at least three identical experiments and expressed as means ± standard deviation. All statistical analyses were carried out using GraphPad Prism (Graph Pad v5 Software, San Diego, CA, USA). Statistical significance of data was evaluated by one-way analysis of variance (ANOVA) followed by the Dunnett’s test or Turkey’s post hoc test. Difference was considered significant when *p*-value was less than 0.05 (*p* < 0.05).

## 3. Results

### 3.1. Anti-Cancer Effects of DIM on Proliferation of HCC Cells

**Figure 2:**
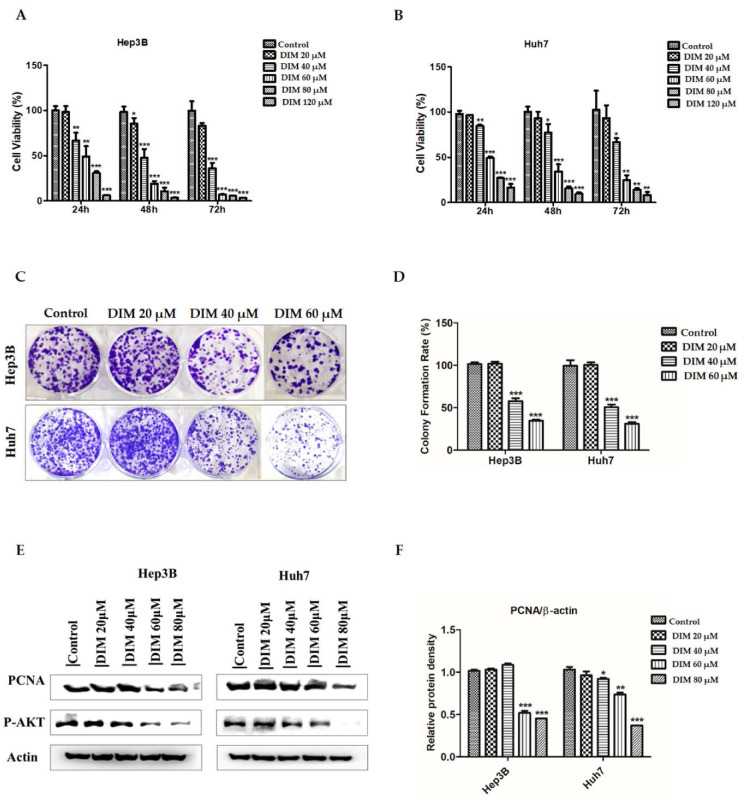
Inhibitory effects of DIM on cell proliferation and related proteins in Hep3B and Huh7 cells. (**A,B**) MTT assay reveals that DIM inhibits cell proliferation of Hep3B and Huh7 HCC cell lines in a dose and time-dependent manner. The bar graph represents the percent proliferation rate compared with the non-treated control. (**C**) Inhibitory effects of DIM on colony formation ability of Hep3B and Huh7 cells. (**D**) Bar graphs indicate quantitative analysis of the number of colonies formed in DIM-treated cells compared to that in the control group of Hep3B and Huh7 cells. (**E**) Western blotting analysis of proliferation-related proteins PCNA and p-AKT in Hep3B and Huh7 cells treated with indicated concentration of DIM for 24 h. (**F**) Bar graphs represent quantification of relative protein expression normalized to β-actin. All data are from three separate experiments (*n* = 3) and expressed as mean ± SE. *, *p* < 0.05; **, *p* < 0.01; and ***, *p* < 0.001, significant difference compared to the control group.

Anti-cancer effects of DIM on HCC cell lines were evaluated with MTT and crystal violet colony formation assays. As shown in Figure 2A,B, DIM treatment significantly reduced cell proliferation of Hep3B and Huh7 cells in a time and dose-dependent manner compared to the untreated control. In addition, DIM shows less toxic effects on the cell viability of normal mouse hepatocytes AML12 as shown in Supplementary Figure S1. Effects of DIM on growth kinetics of Hep3B and Huh7 cells were evaluated by colony formation assay. DIM significantly decreased the number of colonies formed in both Hep3B and Huh7 cells in a concentration-dependent manner compared to the control as shown in Figure 2C,D. Furthermore, DIM significantly reduced expression levels of proliferation cell nuclear antigen (PCNA) and p-AKT in both cell lines as shown in Figure 2E.

### 3.2. DIM Induced Apoptosis of HCC Cells

**Figure 3:**
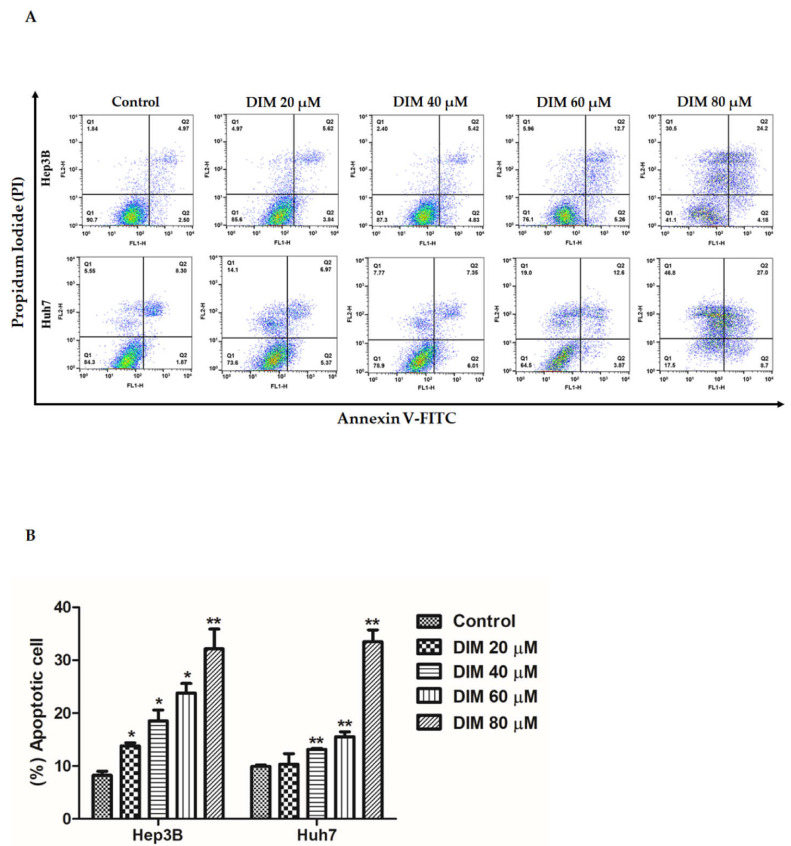
DIM induces apoptosis of HCC cells. (**A**) Flow cytometric analysis of Annexin V-FITC/PI staining in Hep3B and Huh cells after treatment with DIM (0, 20, 40, 60, and 80 µM) for 24 h. (**B**) Bar graphs representing quantitative assessment (**%**) of cell death induced by DIM in Hep3B and Huh7 cells. Data are presented as mean ± SE of three independent experiments. *, *p* < 0.05 and **, *p* < 0.01 denote significant difference compared to the untreated control.

Apoptotic effects of different concentrations of DIM on HCC cells were examined by Annexin V-FITC/propidium iodide staining and flow cytometry analysis. DIM significantly increased the population of apoptotic cells in both early and late-stage compared to the untreated control of both cell lines as shown in Figure 3A,B.

### 3.3. DIM Increases ER Stress-Induced Ca^2+^ Release and Inhibits Mitochondrial Membrane Potential (ΔΨm) to Promote Hepatic Cell Apoptosis

**Figure 4:**
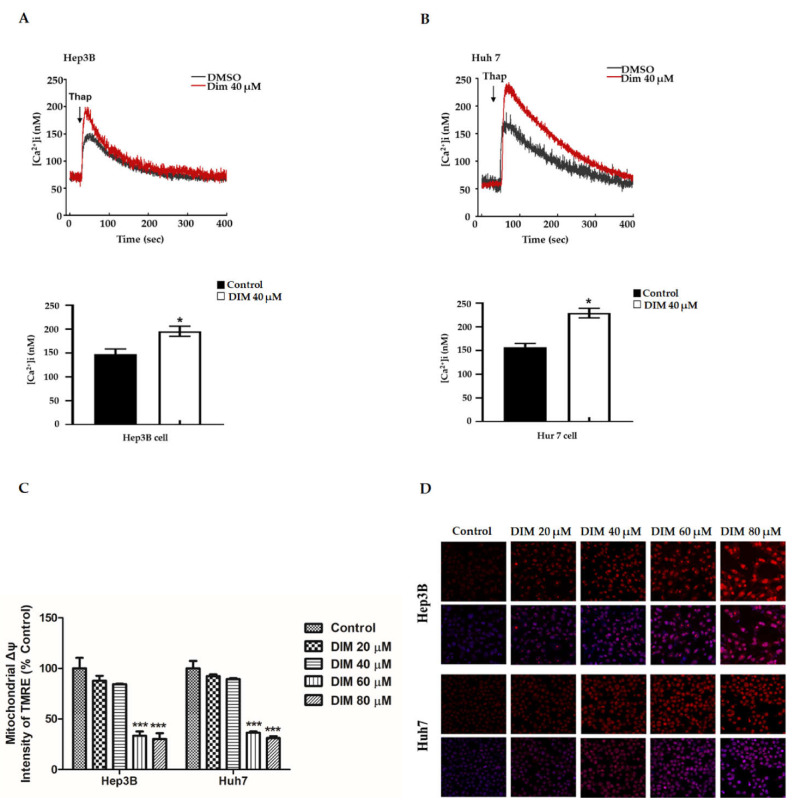

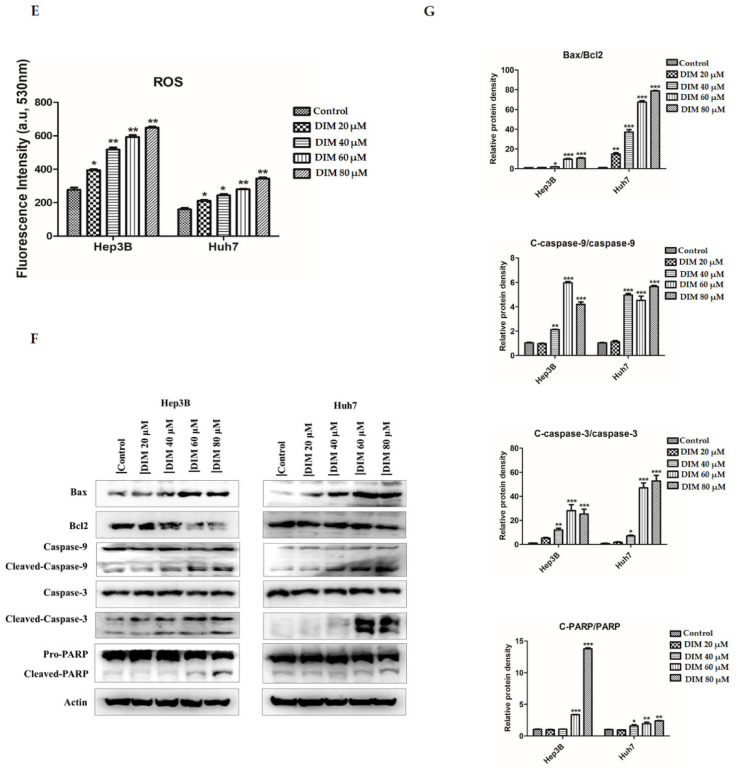
DIM induces apoptosis in HCC cell lines by releasing ER Ca^2+^ and disrupting mitochondrial membrane potential. (**A**,**B**) Respective cell lines and bar graph showing the relative amount of Ca^2+^ flux induced by DIM in Hep3B and Huh7 cells determined by monitoring changes in the Fura-2 ratio upon addition of 1–5 µM thapsigargin using the real-time mode of a PTI system. (**C**) Bar graphs representing quantitative assessment (**%**) for the depletion of mitochondrial membrane potential (ΔΨm) induced by DIM in Hep3B and Huh7 cells using an ELISA reader after staining with tetramethylrhodamine ethyl ester perchlorate (TMRE). (**D**) The amount of intracellular ROS accumulated after DIM treatment in Hep3B and Huh7 cells was evaluated by monitoring the intensity of the ROS fluorescent probe, dihydroethidium (DHE), using a confocal microscope. Scale bar, 30 µm. (**E**) Bar graphs show the amount of ROS induced by DIM treatment in Hep3B and Huh7 cells determined by calculating the relative fluorescence unit (RFU)/µg protein with an Oxi Select In Vitro ROS/RNS Assay Kit according to the manufacturer’s instructions. (**F**) Changes in protein levels of pro-apoptotic and anti-apoptotic related marker induced by DIM treatment as determined by Western blot analysis. (**G**) Quantification of relative protein expression normalized to β-actin. Cells were treated with DIM for 24 h. Provided data represents the mean ± SE of three independent experiments. *, *p* < 0.05; **, *p* < 0.01; and ***, *p* < 0.001 denote significant variation compared to the control group.

The accumulation of calcium from ER stores can induce mitochondrial membrane permeability transition and apoptosis initiating factors from mitochondria [47,48,49]. To determine whether DIM regulated Ca^2+^ distribution, we measured Ca^2+^ release from the ER. To monitor Ca^2+^ levels, Fura-2 Ca^2+^ imaging was performed. Hep3b and Hu7 cells were loaded with a calcium probe Fura-2AM and treated with 1 µM thapsigargin. As shown in Figure 4A,B, 40 µM DIM increased Ca^2+^ in both cell lines as compared with the vehicle control.

Loss of mitochondrial membrane potential due to overproduction of ROS has been reported to play a pivotal role in the initiation of proapoptotic activities [50,51]. Therefore, we investigated effects of DIM on levels of intracellular ROS and ΔΨm using DHE and TMRE specific membrane potential dye. As shown in Figure 4C–E, DIM treatment significantly enhanced the overproduction of intracellular ROS and depletion of mitochondrial membrane potential in both Hep3B and Huh7 cell lines compared with the non-treated control in a concentration-dependent manner. Similarly, to explore whether DIM induced apoptosis, we evaluated expression levels of apoptosis-related proteins in Hep3B and Huh7 cell lines. As shown in Figure 4F,G, immunoblot analysis revealed that DIM treatment significantly increased the expression of cleaved caspase-3, cleaved PARP, cleaved caspase-9, and Bax, but decreased levels of anti-apoptotic proteins Bcl2 in a concentration-dependent order compared to the control. These results suggest that DIM can induce intrinsic apoptosis via ER-stress-induced mitochondrial dysfunction and subsequent cell death as downstream of ROS in HCC cells.

### 3.4. DIM Regulates ER Stress in HCC Cells

**Figure 5:**
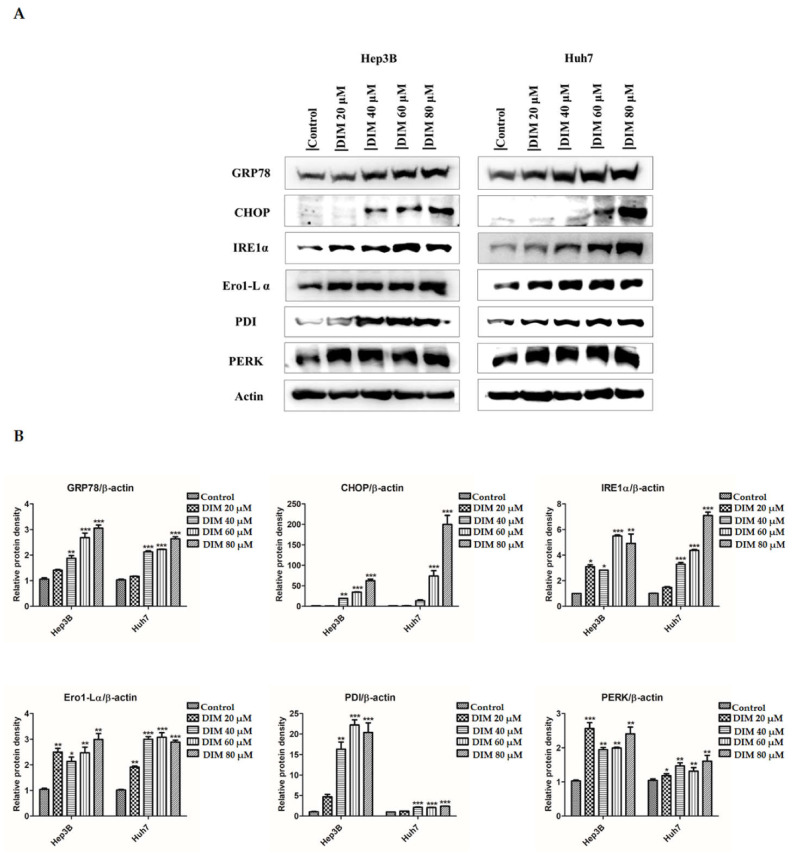

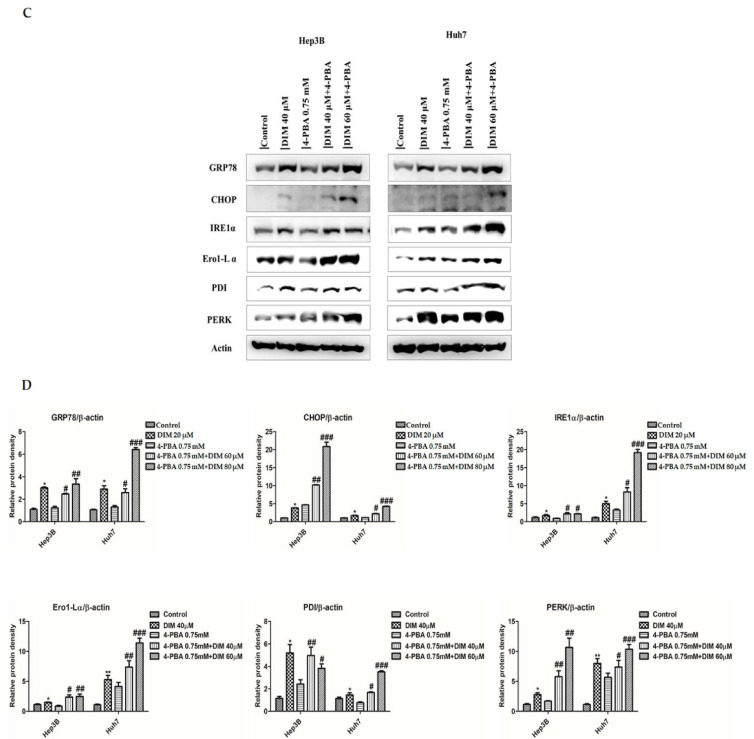
DIM regulates ER stress in HCC cell lines. (**A**) Evaluation of changes of ER stress-related markers at protein level after DIM treatment for 24 h in Hep3B and Huh7 cells by Western blot analysis. (**B**) Bar graphs represents the relative protein expression normalized to β-actin. (**C**) Effect of ER stress inhibitor 4-PBA on DIM-induced ER stress in Hep3B and Huh7 cells based on Western blot analysis. (**D**) Quantification of relative protein expression normalized to β-actin. Provided data represents the mean ± SE of three independent experiments. *, *p* < 0.05; **, *p* < 0.01; and ***, *p* < 0.001 denote significant variation compared to the control group and ^#^, *p* < 0.05; ^##^, *p* < 0.01; and ^###^, *p* < 0.001 denote significant variation compared to the 4-PBA treated group.

ER stress and activation of UPR are known to be involved in the regulation of various cancer cell metabolism. They are considered as unique pathways in regards to the evolution of cancer therapeutic agents [52]. To test the involvement of DIM in the ER stress pathway in HCC cells, we examined the activation of ER stress markers and their downstream signaling molecules by western blot analysis. As shown in Figure 5A,B, DIM treatment significantly increased protein levels of ER-stress related markers CHOP, Bip, Ero-Lα, IRE1α, PERK, and PDI in a dose-dependent order compared to the control. To further determine effects of DIM on ER-stress, the synergistic effect of ER-stress inhibitor 4-PBA and DIM was investigated by Western blot analysis. As shown in Figure 5C,D, ER-stress inhibitor 4-PBA was unable to inhibit the expression of CHOP, Bip, Ero-Lα, IRE1α, PERK, and PDI proteins induced by DIM. These results suggest that DIM can induce ER-stress in Hep3B and Huh7 HCC cells.

### 3.5. DIM Inhibits the Migration and Invasion Potential of HCC Cells

**Figure 6:**
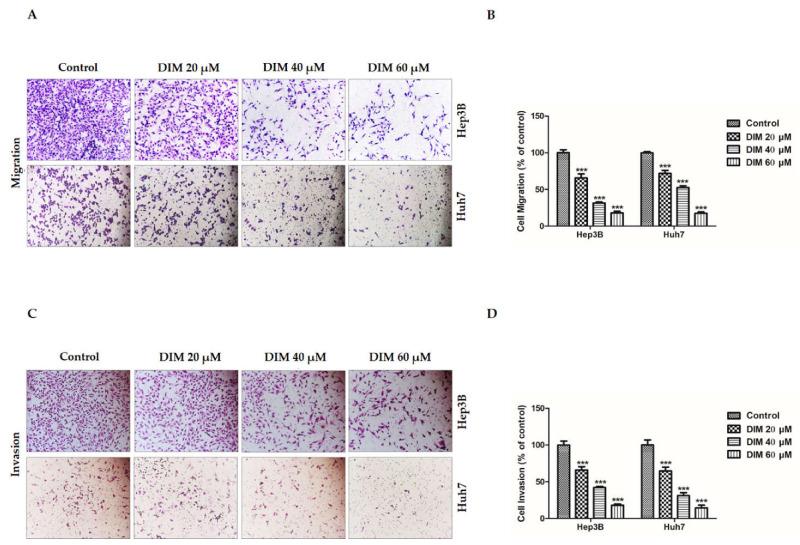
DIM inhibits the migratory and invasive ability of HCC cell lines. (**A**,**C**) Determination of anti-cancer effects of DIM on the migratory and invasive ability of Hep3B and Huh7 cells examined by transwell assays after 24 h treatment with DIM under a phase-contrast microscope at a magnification of ×200. (**B**,**D**) Bar graphs showing the relative assessment (**%**) of numbers of migrated and invaded cells (per unit area) compared to the controls. Data are presented as mean ± SE of three independent experiments. ***, *p* < 0.001 denotes significant variation compared to the control group.

The migration of cancer cells to secondary organs and invasion of the extracellular matrix (ECM) to a secondary organ are essential hallmarks of cancer with a catastrophic role in malignancies and metastasis of various tumors [53]. To determine anti-migratory and anti-invasion potentials of DIM, transwell assays were performed. As shown in Figure 6A,B, Boyden chamber assay revealed that DIM treatment remarkably inhibited the migration ability of Hep3B and Huh7 cells in a dose-dependent manner compared to the non-treated control. Concordantly, Matrigel invasion assays were employed to evaluate the anti-invasive ability of DIM. As shown in Figure 6C,D, DIM drastically reduced the relative number of invaded cells in comparison with the non-treated control. Overall, these results suggest that DIM has an effective anti-migratory and anti-invasive ability to reduce the development of HCC cells.

### 3.6. Anti-Cancer Effects of DIM on the Expression of EMT Markers in HCC Cells

**Figure 7:**
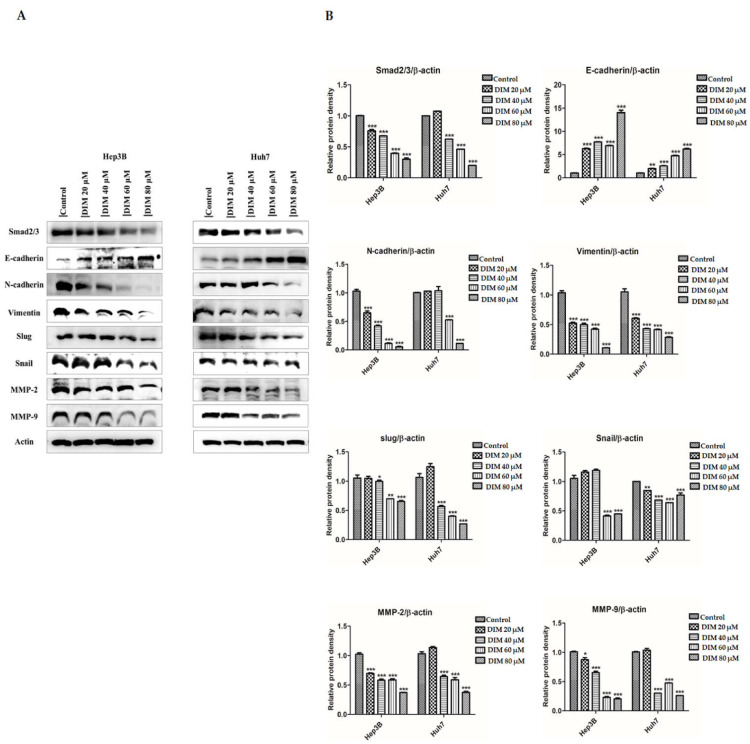
DIM inhibits EMT regulatory proteins in HCC cell lines: (**A**) Anti-cancer effects of DIM on the expression of epithelial–mesenchymal transition (EMT) related protein after 24 h treatment as determined by Western blot analysis. (**B**) Quantification of relative protein expression normalized to β-actin. Provided data represents the mean ± SE of three independent experiments. *, *p* < 0.05; **, *p* < 0.01; and ***, *p* < 0.001 denote significant variation compared to the control group.

EMT is a physio-pathological cellular process that has a crucial role in embryogenesis, wound healing, development of tumor cell metastasis, and drug resistance [54]. To determine the anti-cancer effect of DIM on HCC cells Hep3B and Huh7, expression levels of EMT markers were evaluated. As shown in Figure 7A, DIM treatment significantly enhanced the expression of epithelial target protein E-cadherin, but reduced levels of mesenchymal target proteins such as N-cadherin, vimentin, Snail, and Slug. Besides, DIM treatment remarkably downregulated protein levels of matrix metalloproteinase MMP-2 and MMP-9 in a dose-dependent manner in comparison with the control as shown in Figure 7B, suggesting that DIM could inhibit tumor initiation, invasion, metastasis, and resistance to therapy by suppressing EMT in HCC cells.

### 3.7. DIM Ameliorates 4-PBA-Induced EMT of HCC Cells

**Figure 8:**
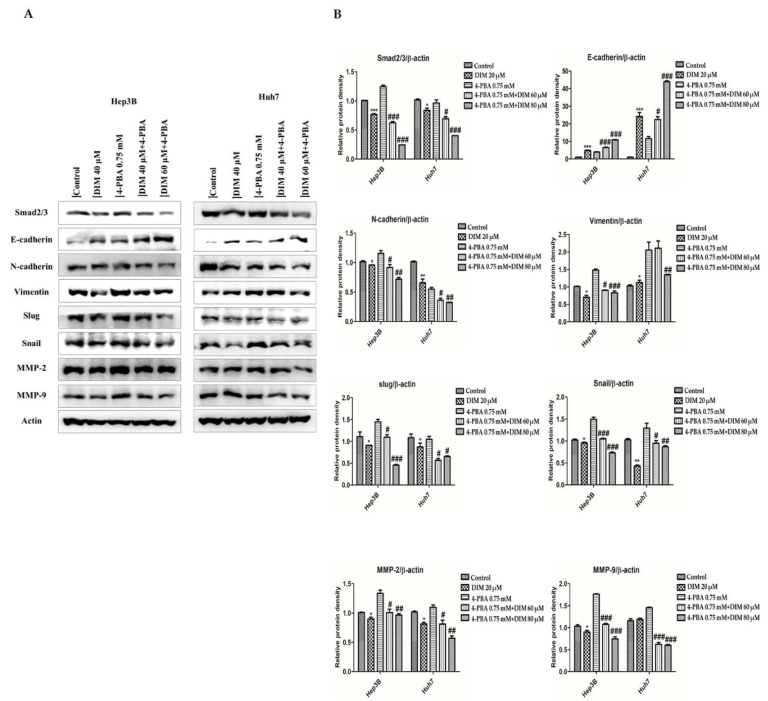
DIM modulates EMT regulatory proteins levels in HCC cell lines. (**A**) Western blot analysis to determine the effect of DIM on the expression of ER-stress regulated EMT proteins levels in Hep3B and Huh7 cells after 24 h DIM treatment. (**B**) Quantification of relative protein expression normalized to β-actin. Provided data represents the mean ± SE of three independent experiments. *, *p* < 0.05; and ***, *p* < 0.001 denote significant variation compared to the control group and ^#^, *p* < 0.05; ^##^, *p* < 0.01; and ^###^, *p* < 0.001 denote significant variation compared to the 4-PBA treated group.

EMT plays a crucial role in the activation of the invasion-metastasis cascade in various tumors by regulating ER stress and the UPR signaling pathways [55]. To determine effects of DIM on ER-stress mediated EMT, both Hep3B and Huh7 cells were treated with a combination of DIM and a classic ER-stress inhibitor 4-phenylbutyric acid (4-PBA). Protein levels of EMT and matrix metalloproteinase related proteins were then determined. As shown in Figure 8A, 4-PBA (0.75 mM) was unable to inhibit the expression of epithelial target proteins E-cadherin induced by DIM compared to treatment with 4-PBA alone which similarly failed to restore proteins levels of mesenchymal target markers such as N-cadherin, vimentin, slug, and snail inhibited by DIM treatment. Furthermore, 4-PBA was unable to enhance the expression of matrix metalloproteinase MMP-2 and MMP-9 inhibited by DIM compared to those treated with 4-PBA alone as shown in Figure 8B. These data suggest that the anti-tumor effect of DIM is mediated by inhibition of ER-stress mediated EMT of Hep3B and Huh7 cells.

## 4. Discussion

HCC is still considered one of the most dreadful diseases that can cause an increased incidence of intrahepatic and extrahepatic metastasis with a high mortality rate in both developed and developing countries [1,56]. Due to its poor prognosis and lack of management with adjuvant therapies, the only treatment for HCC is hepatic resection and liver transplantation with the minimum outcome, systemic toxicity, distant metastasis, and drug resistance. Therefore, a specific, efficient, and non-toxic novel therapeutic agent against HCC is highly desirable. Epidemiologic evidence has suggested that many natural and dietary phytochemicals extracted from medicinal plants possess therapeutic potential against cancer development and can inhibit carcinogenesis both in vitro and in vivo [57,58,59]. 3,3′-Diindolylmethane (DIM) is a dietary phytochemical extracted from bioactive condensation of indole-3-carbinol (I3C) found in cruciferous vegetables. It shows a variety of biological and biochemical effects [36,60]. Emerging evidence had suggested that DIM exhibits chemopreventive and growth-inhibiting potential in various cancers—such as prostate cancer [61,62], breast cancer [63], pancreatic cancer [64,65], liver cancer [35,66,67], and esophageal cancer [39]—by regulating multiple oncogenic signaling pathways. However, there are no significant shreds of evidence reported for the anti-cancer role of DIM by regulating ER stress-mediated mitochondrial dysfunction and apoptotic death. The present study revealed that DIM exhibited an anti-cancer effect by inducing ER stress, apoptosis, and inhibiting invasion and migration of hepatocarcinoma cells, suggesting that DIM could be a potential anti-cancer agent for treating cancers.

The endoplasmic reticulum (ER) is an intracellular organelle that plays an important role in regulating cellular homeostasis and toxins-induced cell death [68]. ER activity can be disrupted by different physiological and pathological conditions, causing the accumulation of unfolded proteins that can induce ER stress and lead to the activation of UPR and the initiation of apoptosis. In mammalian cells, the activation of UPR by ER stress is regulated by three ER transmembrane proteins: ATF6, PERK, and IRE1α [69]. Emerging evidence suggests that phytochemicals product may exert a potential effect against cancer, metabolic diseases, cardiovascular diseases, and neurodegenerative diseases such as Alzheimer’s disease and Parkinson’s disease by regulating ER stress signaling pathway [70,71]. Concordantly, our study showed that DIM pretreatment significantly increased the expression of ER stress-related proteins Bip (GRP78), CHOP, PERK, IRE1α, Ero1-Lα, and PDI in a dose-dependent manner compared with the control in both Hep3B and Huh7 cells. Furthermore, numerous studies have suggested that addition of ER-stress inhibitor reduces the expression of CHOP, in ER stress and reduces ER-stress-induced apoptosis [43,72,73]. Consistently, in the present study, the addition of a potent ER stress inhibitor 4-PBA known to act as a chemical chaperone to inhibit UPR signaling was unable to rectify the expression of ER stress-related proteins induced by DIM compared to treatment with 4-PBA alone. These results suggest that DIM can induce ER stress-mediated UPR in HCC cells.

ER can act as an intracellular store for calcium ions (Ca^2+^) and lead to calcium homeostasis which is important for maintaining normal cell function. In response to various pathophysiological stress and stimuli, ER stress can disrupt ER Ca^2+^ stores and lead to the depletion or release of Ca^2+^ from ER to cytosol, causing growth arrest and cell death [74]. Calcium can act as a key regulator in mitochondria to stimulate ATP synthesis. Previously, numerous studies have suggested that efflux of Ca^2+^ from ER to mitochondria can result in mitochondrial dysfunction, causing overproduction of ROS, which in turn results in cell death [75,76]. Several studies have reported that overproduction of mitochondrial ROS can result in mitochondrial membrane depolarization, causing a loss in mitochondrial membrane potential which initiates mitochondrial-dependent apoptotic pathway [4,77]. Although many studies have investigated the relationship among ROS, Ca^2+^, and apoptosis in several cancer cell lines [78,79], little is known about the role of mitochondria, ROS, and Ca^2+^ in the induction of apoptosis caused by DIM. In the present study, we found that DIM treatment increased the efflux of ER Ca^2+^ to mitochondria with or without ER stress agents thapsigargin (TG), enhancing the overproduction of ROS and leading to the depletion of mitochondrial membrane potential, which in turn caused apoptosis of both Hep3B and Huh7 cells. Taken together, these results provide novel evidence about the mechanism involved in DIM-induced ROS and mitochondrial dysfunction or ER stress pathway.

Apoptosis is termed as programmed cell death that is important for maintaining cellular homeostasis by eliminating dysfunctional and injured cells. It is also considered as a major route for anti-cancer compounds to suppress cancer cell proliferation [80]. Apoptosis is regulated by two main pathways: the intrinsic (mitochondria-mediated) pathway and the extrinsic pathway. The intrinsic pathway is characterized by the loss of mitochondrial membrane potential, the release of cytochrome C from mitochondria to cytosol, and the activation of caspase-The extrinsic pathway is featured by an auto-catalytic activation of caspase-8 [81]. In the present study, we found that DIM-induced apoptosis was associated with the activation of the intrinsic apoptotic pathway that involved the activation of pro-apoptotic proteins Bax. DIM also reduced the expression of anti-apoptotic proteins Bcl-It is well known that the expression of CHOP and PERK is activated by a disturbance in ER homeostasis. Accumulating evidence has suggested that activation of CHOP can initiate apoptosis and inhibit the expression of Bcl-2 protein [82]. Concordantly, in the present study, we found that DIM mediated apoptosis in both Hep3B and Huh7 cells by inducing the expression of CHOP which in turn activated the expression of pro-apoptotic proteins Bax and inhibited anti-apoptotic proteins Bcl-DIM also induced the expression of classical apoptotic related proteins such as cleaved caspase-3, cleaved caspase-9, and cleaved PARP. Taking together, our results suggest that DIM-induced apoptosis is regulated by the activation of the intrinsic apoptotic signaling pathway.

EMT plays an important role in the progression of cancer cell invasion and migration by losing epithelial cell polarity and reducing cell ability to bind to the basement membrane to transform into mesenchymal stem cells [4,83]. In tumor biology, the EMT process is involved in malignancies of tumor cells and the progression of metastasis [54]. During the activation of the EMT process, cancer cells can shed their epithelial characteristics and become invasive by inducing the expression of EMT transcriptional factors such as Slug, Snail, and Smad 2/3 and mesenchymal markers like α-SMA, N-cadherin, and Vimentin while repressing the expression of epithelial markers epithelial cadherin (E-cadherin) and zonula occludens-1 (ZO-1) [84]. Consistently, in our study, we found that DIM treatment significantly inhibited EMT by restoring protein levels of E-cadherin while decreasing the expression of N-cadherin, Vimentin, Snail, and Slug in both Hep3B and Huh7 cell lines. Degradation of the extracellular matrix (ECM) has been linked to the invasive nature of malignant tumors [85]. Matrix metalloproteinases (MMPs) are a family of zinc-dependent endopeptidases involved in the degradation of ECM components and regulation of tumor metastatic cascade [86]. In HCC, MMP-2 and MMP-9 are two main components of MMPs that play an important in cancer cell invasion and migration [87]. Previous studies have shown the potential effect of DIM against cancer migration by downregulating MMPs in several cancers, including breast cancer [88], thyroid cancer [89], and prostate cancer [41]. Consistently, in our study, we found that DIM treatment significantly decreased the expression of MMP2/9 in both Hep3B and Huh7 cell lines. Furthermore, DIM treatment inhibited migration and invasion rates of both Hep3B and Huh7 cell lines, confirming the anti-metastatic role of DIM. Emerging evidence has suggested that activation of UPR plays a crucial role in the regulation of tumor cell migration and metastasis [90]. However, a recent study has shown that ER stress can induce EMT by regulating Smad2 and Src pathways in alveolar epithelial cells [91]. PERK and XBP1 are the main branch of ER membrane proteins that can modulate tumor cell migration and invasion features such as ECM and EMT [30,92]. For example, overexpression of XBP1 promotes the invasive and metastatic potential and regulates EMT in HCC cells [30]. In the present study, we found that overexpression of EMT and MMPs markers induced by ER stress-specific inhibitor 4-PBA was repressed by DIM treatment, suggesting that DIM could attenuate ER stress-induced EMT in HCC cell lines.

Taken together, our results suggest that DIM can significantly inhibit cell proliferation, migration, and invasion while inducing the apoptosis of HCC cell lines. Furthermore, we found that DIM treatment resulted in an overproduction of ROS that caused loss of mitochondrial membrane potential and triggered mitochondrial dependent apoptotic pathway in HCC cells. DIM treatment also regulated ER stress, UPR activation, and Ca^2+^ release, which activated the caspase cascade, ultimately initiating apoptosis. Our results suggest that the molecular mechanism involved in the anticancer activity of DIM could lead to the development of novel and promising treatment for hepatocellular carcinoma.

## 5. Conclusions

**Figure 9:**
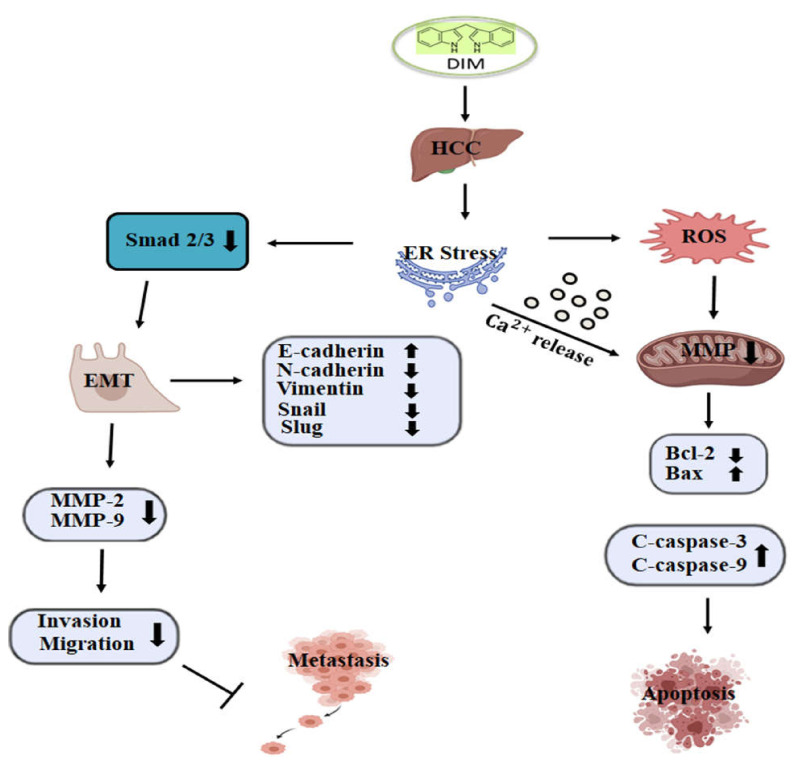
Graphical representation of proposed mechanisms by which DIM induces apoptosis and inhibits metastasis in HCC. DIM induces ER stress, accumulation of ROS, and Ca^2+^ release into the cytosol, which acts on the mitochondria and causes a loss of mitochondrial membrane potential, leading to the activation of hepatic cell death. DIM-mediated ER stress also modulates the Smad2/3 pathway, inhibits EMT, and reduces cell invasion and migration ability, thus suppressing cancer metastasis.

In conclusion, our study demonstrated the anti-cancer ability of DIM in suppressing the growth of HCC by inhibiting cell proliferation, migration, and invasion and by inducing apoptosis. DIM induced ER stress and activated UPR response, leading to the expression of key indicators such as Bip, IRE1α, CHOP, and PDI. Furthermore, DIM caused the release of Ca^2+^ from ER into the cytoplasm that resulted in an overproduction of ROS and a depletion of mitochondrial membrane potential, leading to the activation of the intrinsic apoptotic pathway. Additionally, DIM inhibited tumor growth and metastasis of Hep3B and Huh7 cells by modulating ER stress mediated Smad 2/3 pathway to inhibit EMT, thus preventing migration and invasions (Figure 9). These results suggest that the pharmacological mechanism of DIM might lead to a potential therapeutic anti-cancer therapy for HCC.

## Figures and Tables

**Table 1:** List of specific primary antibodies and secondary antibodies used in Western blotting.

S.N	Target	Blocking Solution	Dilution	Secondary	Manufacturer	Catalogue Number
1	PCNA	5% Skim Milk	1:3000	Rabbit IgG	Santa Cruz	7907
2	P-AKT	5% Skim Milk	1:2000	Rabbit IgG	Cell Signaling	4060
3	β-actin	5% Skim Milk	1:3000	Mouse IgG	Sigma Aldrich	A5441
4	Cleaved-Caspase-3	5% BSA	1:2000	Rabbit IgG	Cell Signaling	9661
5	Cleaved-Caspase-9	5% BSA	1:2000	Rabbit IgG	Cell Signaling	20,750
6	Cleaved-PARP	5% BSA	1:2000	Rabbit IgG	Cell Signaling	5625
7	Bax	5% Skim Milk	1:3000	Rabbit IgG	Santa Cruz	493
8	Bcl-2	5% BSA	1:3000	Rabbit IgG	BioWorld	1511
9	E-cadherin	5% BSA	1:2000	Rabbit IgG	Cell Signaling	3195
10	N-cadherin	5% BSA	1:2000	Mouse IgG	BD Biosciences	610,920
11	Vimentin	5% BSA	1:1000	Rabbit IgG	Cell Signaling	5741
12	Snail	5% BSA	1:1000	Rabbit IgG	Cell Signaling	3879
13	Slug	5% BSA	1:1000	Rabbit IgG	Cell Signaling	9585
14	MMP-2	5% BSA	1:3000	Rabbit IgG	BioWorld	1236
15	MMP-9	5% BSA	1:1000	Rabbit IgG	Cell Signaling	2270
16	Bip	5% BSA	1:3000	Rabbit IgG	Cell Signaling	3177
17	Ero1-Lα	5% BSA	1:1000	Rabbit IgG	Cell Signaling	3264
18	IRE1α	5% BSA	1:1000	Rabbit IgG	Cell Signaling	3294
19	PDI	5% BSA	1:1000	Rabbit IgG	Cell Signaling	3501
20	CHOP	5% BSA	1:1000	Rabbit IgG	Cell Signaling	2895
21	PERK	5% BSA	1:1000	Rabbit IgG	Cell Signaling	5683
22	Smad 2/3	5% BSA	1:1000	Mouse IgG	BD Biosciences	610,842

(BSA: bovine serum albumin); (Santa Cruz Biotechnology, Inc. Delaware, CA, USA; Cell signaling Technology, Danvers, MA, USA; Sigma Aldrich, St. Louis, MO, USA; BioWorld Technology, Inc. Bloomington, MN, USA; BD biosciences Pharmingen, San Diego, CA, USA).

## Data Availability

Not applicable.

## Supplementary Materials

The following are available online at https://www.mdpi.com/article/10.3390/cells10051178/s1, Figure S1: Effects of DIM on the cell viability of normal hepatocyte AML12 cells.
