# Supplementary material for: 3,3′-Diindolylmethane Suppresses the Growth of Hepatocellular Carcinoma by Regulating Its Invasion, Migration, and ER Stress-Mediated Mitochondrial Apoptosis
Source: Cells. 2021 May 12;10(5):1178. doi: 10.3390/cells10051178 (PMC8151225; doi:10.3390/cells10051178)
Supplement: Supplementary file 1 [file cells-10-01178-s001.zip › cells-1178882-supplementary.pdf]

cells

MDPI

Supplementary Figure S1

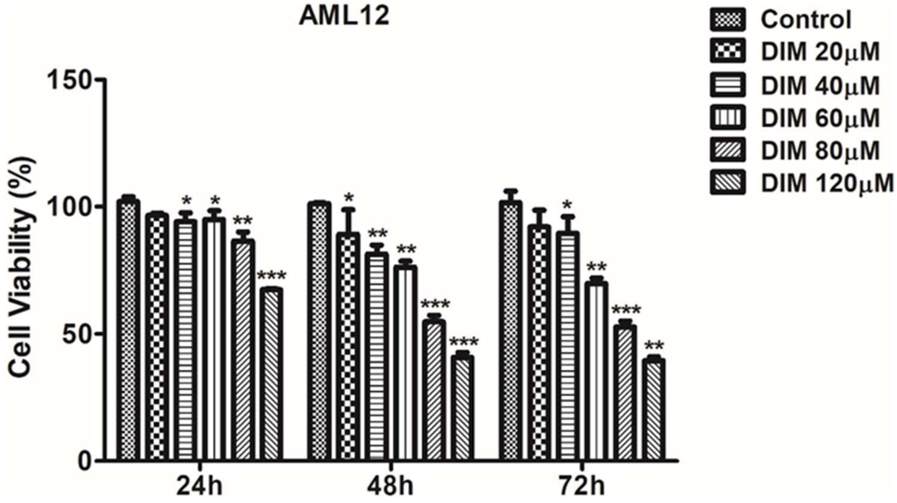

Figure S1. Effects of DIM on the cell viability of normal hepatocytes AMl12. MTT assay evaluated the toxicity of DIM in the cell viability of AML12 cells. All data are from three separate experiments ($n = 3$) and expressed as mean ± SE. * $p < 0.05$; ** $p < 0.01$, and *** $p < 0.001$, significant difference compared to the control group.
